# *In ovo* leptin administration affects hepatic lipid metabolism and microRNA expression in newly hatched broiler chickens

**DOI:** 10.1186/2049-1891-3-16

**Published:** 2012-06-01

**Authors:** Yan Hu, Rui Zhang, Yanhong Zhang, Jing Li, Roland Grossmann, Ruqian Zhao

**Affiliations:** 1 Key Laboratory of Animal Physiology & Biochemistry, Nanjing Agricultural University, Nanjing, 210095, China; 2 Key Laboratory of Poultry Heredity & Breeding, Institute of Poultry Science of Jiangsu Province, Yangzhou, 225003, China; 3 Department of Functional Genomics & Bioregulation, Institute of Animal Science Mariensee, 31535, Neustadt, Germany

**Keywords:** Broiler chickens, *In ovo* manipulation, Leptin, Lipid metabolism, Liver, MicroRNA, SREBPs

## Abstract

**Background:**

A leptin-like immunoreactive substance has been found in chicken eggs and has been implicated in serving as a maternal signal to program offspring growth and metabolism. In the present study, we investigated the effects of *in ovo* leptin administration on hatch weight, serum and hepatic concentrations of metabolites and hormones, as well as on the expression of genes involved in hepatic lipid metabolism and the predicted microRNAs (miRNAs) targeting the affected genes. To this end we injected fertile eggs with either 0.5 μg of recombinant murine leptin or vehicle (PBS) before incubation.

**Results:**

Prenatally leptin-exposed chicks showed lower hatch weight, but higher liver weight relative to the body weight, compared to the control group. *In ovo* leptin treatment increased the hepatic content and serum concentration of leptin in newly hatched chickens. The hepatic contents of triglycerides (TG) and total cholesterol (Tch) were decreased, whereas the serum levels of TG, Tch and apolipoprotein B (ApoB) were increased. The hepatic mRNA expression of *sterol regulator element binding protein 1* (*SREBP-1c*), *SREBP-2*, *hydroxy-3-methylglutaryl coenzyme A reductase* (*HMGCR*) and *cholesterol 7α-hydroxylase 1* (*CYP7A1*) was significantly up-regulated, as was the protein content of both SREBP-1c and SREBP-2 in hepatic nuclear extracts of leptin-treated chickens. Moreover, out of 12 miRNAs targeting *SREBP-1c* and/or *HMGCR*, five were significantly up-regulated in liver of leptin-treated chicks, including *gga-miR-200b* and *gga-miR-429*, which target both *SREBP-1c* and *HMGCR*.

**Conclusions:**

These results suggest that leptin *in ovo* decreases hatch weight, and modifies hepatic leptin secretion and lipid metabolism in newly hatched broiler chickens, possibly via microRNA-mediated gene regulation.

## Background

Leptin is involved in the regulation of food intake and energy balance in mammals [1,2]. Despite the current controversy over the existence of a *leptin* gene in the chicken genome [3], the existence of a leptin-like immunoreactive substance [4,5] and a functional leptin receptor (LEPR) has been confirmed [6]. Furthermore, exogenous murine or human leptin exerts similar effects on poultry as it does on mammals [7,8].

Maternal leptin has been shown to program offspring obesity in mammals [9]. Fetal or neonatal abnormal nutritional environment induces leptin synthesis and secretion from adipocytes, and affects adipocyte morphology and metabolism, thus linking embryonic nutrition to adult obesity [10]. Manipulating either maternal plasma leptin or transplacental leptin transfer could impact the postnatal regulation of leptin synthesis and secretion in the offspring [11-13]. Previously, we have demonstrated the presence of a leptin-like immunoreactive substance in the yolk and albumen of chicken eggs [4]. Moreover, feeding hens with low-protein [14] or cysteamine-supplemented diets [4] affects leptin deposition in eggs, as well as early posthatch growth and metabolism of chickens. *In ovo* administration of leptin improved embryonic development and resulted in higher body weight at hatching of Japanese quails [15]. These findings imply possible roles of maternal leptin deposited in the egg in programming offspring growth and metabolism in poultry.

Leptin is mainly secreted by mammalian adipose tissue, and exerts direct effects on lipolysis and lipogenesis of adipose tissue [2]. In birds, liver is the major source of leptin [16] and the primary organ for lipogenesis [17]. Therefore, it is of interest whether *in ovo* administration of leptin affects hepatic leptin synthesis and secretion, as well as hepatic lipid homeostasis in newly hatched chickens.

Hepatic lipid homeostasis is regulated by a family of membrane-bound transcription factors, designated sterol regulatory element binding proteins (SREBPs) [18]. SREBP-1c, the most abundant isoform of SREBP-1 in the liver, preferentially enhances transcription of enzymes required for fatty acid synthesis, including acetyl CoA carboxylase (ACC), carnitine palmitoyltransferase I (CPT-I) and fatty acid synthase (FAS), whereas SREBP-2 is more selective for activating genes involved in cholesterol homeostasis, including HMG-CoA reductase (HMGCR) and cholesterol 7α-hydroxylase 1 (CYP7A1) [18]. Leptin has been shown to negatively regulate the expression of SREBPs [2], HMGCR [19], CYP7A1 [19], ACC and FAS [2]*,* thus inhibiting lipogenesis in mammalian species.

MicroRNAs (miRNAs) are small noncoding RNAs with an average size of 22 nt. They bind to complementary sequences of target messenger RNA transcripts (mRNAs), usually resulting in translational repression and gene silencing [20]. A number of mammalian miRNAs that execute post-transcriptional regulation of genes involved in lipid homeostasis have been identified [21,22]. Recently, *miR-33*, which is located in the intron of SREBPs, was reported to regulate cholesterol metabolism [23,24]. Moreover, miRNAs have been shown to mediate the effects of leptin on adipocyte differentiation [25] and adipogenesis [26] in mammals. However, it is unknown whether the programming effects of maternal leptin involve miRNA-mediated post-transcriptional regulation. Relatively little is known about the function of miRNAs in chickens. More than 50 miRNAs have been identified in chicken liver [27-29], but only *miR-33* has been experimentally verified to target the *FAS* gene in chicken fibroblast cells [30]. A correlation study linking the expression of miRNAs and their target genes, in association with hepatic lipid homeostasis may shed light on the potential functions of miRNAs in the chicken liver.

Therefore, the present study aimed to investigate the effects of *in ovo* leptin administration on hepatic leptin synthesis and secretion, as well as on hepatic lipid homeostasis in newly hatched chickens. The expression of genes involved in hepatic lipid metabolism, such as *SREBP*s and *HMGCR*, and of the predicted miRNAs targeting the relevant genes was also determined to reveal the possible mechanisms.

## Methods

### Animals and experimental design

Fertile breeder eggs purchased from Sanhuang broiler breeding farm (Wen’s group, Guangdong, China) were randomly divided into two groups and injected with 0 μg (Control, Con) or 0.5 μg (Leptin, Lep) of recombinant murine leptin (498-OB-01 M, R&D, Minneapolis, MN, USA) in 100 μL of phosphate buffered saline (PBS) before incubation. After injection, all eggs were incubated in a forced draught incubator with automatic turning every two hours at 37.5 ± 0.3°C and 50% to 60% humidity. At hatching (D0), chicks from each group were weighed and 12 chicks (6 males and 6 females) from each group were euthanized to take blood and liver samples. All tissue samples were snap frozen in liquid nitrogen and stored at -80°C. The experiments were undertaken following the guidelines of the Animal Ethics Committee of Nanjing Agricultural University, China.

### Measurement of lipid parameters

Total lipid content in homogenized liver samples was extracted using a mixture of chloroform and methanol (2:1 v/v) according to a previously reported method [31]. The hepatic contents and serum concentrations of triglycerides (TG) and total cholesterol (TC) were determined using commercial kits (GPO-PAP and CHOD-PAP) purchased from Nanjing Jiancheng Biotechnology Institute (NJBI, Nanjing, Jiangsu, China) following the manufacturer’s protocols.

### Tissue extraction and measurement of leptin and apolipoprotein B

Frozen liver samples (approximately 200 mg) were homogenized in 2 mL of ice-cold lysis buffer (50 mmol/L Tris-HCl, pH 7.5, 0.2% Triton X-100 and protease inhibitor mixture) using a tissue grinder (Polytron PT1200E, Brinkman Instruments, Littau, Switzerland). The homogenate was then centrifuged at 5,000 × g for 20 min at 4°C to remove all insoluble material. The supernatant was collected, and the protein concentration was determined with a Bradford assay kit purchased from NJBI.

Leptin in serum and liver extracts was measured with a commercial multi-species RIA kit purchased from Beijing North Institute of Biotechnology (Beijing, China). The detection limit for leptin was 0.45 ng/mL. The intra- and inter-assay coefficients of variation were 5% and 10%, respectively. The commercial RIA kit was previously validated for measuring chicken samples [32]. Serum apolipoprotein B (ApoB) was determined using a commercial kit purchased from Biosino Biotechnology Company Ltd. (Beijing, China).

### Western blot analysis

Nuclear and cytoplasmic protein was extracted from liver samples using Nuclear Protein Extraction Kit (PR116, Yuanpinghao Biotechnology Ltd., Beijing, China). Nuclear protein extracts were used for detecting SREBP-1c and SREBP-2, while cytoplasmic protein extracts were used for determining HMGCR and CYP7A1 levels. Whole cell lysates were prepared for quantitating leptin receptor (LEPR) by western blot analysis.

After electrophoresis, proteins were transferred to nitrocellulose membranes, which were then blocked with 5% fat-free milk or 3% BSA in Tween-Tris-buffer saline (TBST) for 2 h at room temperature. After repeated washing with TBST, the membranes were incubated with monoclonal antibodies against LEPR (diluted 1:1,000; a generous gift from Dr. Ohkubo, Faculty of Agriculture, Kagawa University, Japan), SREBP-1c (MA1-38651, Thermo, Waltham, MA, USA; diluted 1:200), SREBP-2 (ab30682, Abcam, UK; diluted 1:300), HMGCR (diluted 1:1,000; sc-33827, Santa Cruz, California, CA, USA) or CYP7A1 (diluted 1:200; ab79847, Cambridge, Abcam), followed by goat polyclonal horseradish peroxidase conjugated secondary antibody to rabbit IgG (diluted 1:5,000; ab6721, Abcam) or mouse IgG (diluted 1:4,000; GGHL-90P, Immunology Consultants Laboratory, Newberg, OR, USA), as previously described [6]. Finally, the membranes were washed and the specific signals were detected by chemiluminescence using the LumiGlo substrate (Super Signal West Pico Trial Kit, Pierce, Rockford, IL, USA). Enhanced chemiluminescence (ECL) signals recorded on x-ray film were scanned and analyzed with Kodak 1D Electrophoresis Documentation and Analysis System 120 (Kodak Photo Film Co. Ltd., Rochester, NY, USA). The membranes were stripped and reprobed with an antibody against β-actin (diluted 1:4,000; ab8227, Abcam) or LAMINA/C (diluted 1:500; BS1446, Bioworld Technology, Minneapolis, MN, USA), followed by horseradish peroxidase conjugated secondary antibodies (diluted 1:5,000; ab6721, Abcam). β-actin was used to normalize the band density of LEPR, HMGCR and CYP7A1, and LAMINA/C was used to normalize the band density of SREBP-1c and SREBP-2. The hepatic contents of LEPR, SREBP-1c, SREBP-2, HMGCR and CYP7A1 were presented as the fold change relative to the average values of the control group.

### RNA extraction and mRNA quantification

Total RNA was extracted using TRIzol total RNA Kit (Tiangen Biotech Co., Ltd., Beijing, China), and 2 μg of total RNA were reverse-transcribed in a final volume of 25 μL with M-MLV reverse transcriptase (M1701, Promega, Madison, WI, USA) in a Bio-Rad DNA Engine Peltier Thermal Cycler PTC0200 (Bio-Rad, Hercules, CA, USA). Real-time PCR was performed to quantitate *LEPR*, *SREBP-1c/2*, *HMGCR* and *CYP7A1* mRNA with Mx3000P (Stratagene, Santa Clara, CA, USA). The primers were designed and synthesized by TaKaRa Biotechnology Co., Ltd. (Dalian, Shandong, China; Table 1).

**Table 1:** Primer sequences for the target genes

Target genes	GenBank accession numbers	PCR products (bp)	Primer sequences
*β-actin*	NM 205518	300	F: 5′- tgcgtgacatcaaggagaag -3′
			R: 5′- tgccagggtacattgtggta -3′
*LEPR*	NM 204323	87	F: 5′- gcatctctgcatctcaggaaaga -3′
			R: 5′- gcaggctacaaactaacagatcca -3′
*SREBP-1c*	NM 204126	104	F: 5′- gcagaagagcaagtccctcaa -3′
			R: 5′- tcggcatctccatcacctc -3′
*SREBP-2*	XM 416222	108	F: 5′- cccagaacagcaagcaagg -3′
			R: 5′- gcgaggacaggaaagagagtg -3′
*HMGCR*	AB 109635	137	F: 5’-ttggatagagggaagagggaag -3’
			R: 5’- ccatagcagaacccaccaga-3’
*CYP7A1*	AB 109636	106	F:5’- cattctgttgccaggtgatgtt -3’
			R:5’- gctctctctgtttcccgcttt -3’

### Real-time PCR

Different controls were set to monitor the possible contamination of genomic and environmental DNA both at the stage of reverse transcription and PCR. A pooled sample made by mixing an equal quantity of total RNA from all samples was used for optimizing the PCR conditions and tailoring the standard curves. Two microliters of 8-64-fold dilutions of each reverse transcription product were used for PCR in a final volume of 25 μL. The PCR end products were verified with the melting curves that showed a single peak specific for the target gene.

### miRNA quantification

Total RNA isolated from liver was treated with RNase-free DNase I (TaKaRa, Tokyo, Japan), and then polyadenylated (2 μg) by poly(A) polymerase (Ambion, Austin, TX, USA) at 37°C for 1 h in a 20 μL reaction mixture. After phenol-chloroform extraction and isopropyl ethanol precipitation, the RNA was dissolved and reverse-transcribed using a poly(T) adapter [33]*.*

qPCR was performed using SYBR Green Real-time PCR Master Mix (TaKaRa) with a miRNA-specific forward primer and a universal reverse primer complementary to part of the poly(T) adapter sequence. Because no validated reference gene was available for chicken miRNA, random DNA oligonucleotides were added to the RNase-free Dnase I-treated total RNA samples before polyadenylation, as an exogenous reference, to normalize the expression of miRNAs. The sequences for all the primers, poly(T) adapter and the exogenous reference gene used are listed in Table 2.

**Table 2:** Primer sequences for miRNAs used in the study

Names	Primer sequences
gga-miR-15a	F: 5′- TAGCAGCACATAATGGTTTGT -3′
gga-miR-15b	F: 5’- TAGCAGCACATCATGGTTTGCA -3’
gga-miR-16	F:5’- TAGCAGCACGTAAATATTGGTG -3’
gga-miR-27b	F:5’- TTCACAGTGGCTAAGTTCTGC -3’
gga-miR-99a	F:5’- AACCCGTAGATCCGATCTTGTG -3’
gga-miR-100	F: 5′- AACCCGTAGATCCGAACTTGTG -3′
gga-miR-181a	F: 5′- AACATTCAACGCTGTCGGTGAGT -3′
gga-miR-183	F: 5′- AACATTCATTGCTGTCGGTGGG -3′
gga-miR-200a	F: 5′- TAACACTGTCTGGTAACGATGT -3′
gga-miR-200b	F: 5′- TAATACTGCCTGGTAATGATGAT -3′
gga-miR-223	F: 5′- TGTCAGTTTGTCAAATACCCC -3′
gga-miR-429	F: 5′- TAATACTGTCTGGTAATGCCGT -3′
exogenous reference	F: 5′- GTGACCCACGATGTGTATTCGC -3′
universal reverse primer	F: 5′- TAGAGTGAGTGTAGCGAGCA -3′
poly(T) adapter	F:5′- TAGAGTGAGTGTAGCGAGCACAGAATTAATACGACTCACTATAGG(T)_16_VN-3′

No 5′UTR sequence was available for the chicken *CYP7A1* gene, therefore we just predicted the miRNAs targeting *SREBP-1cSREBP-2* and *HMGCR* with Targetscan 5.1 (http://www.targetscan.org/) [34-36]. Five miRNAs were predicted to target *SREBP-1c*, while nine miRNAs were predicted to target *HMGCR*. Only one miRNA, *gga-miR-138*, was predicted to target *SREBP-2*. However, we were unable to amplify specific *gga-miR-138* from the chicken hepatic samples; therefore we excluded *gga-miR-138* from the present study.

### Statistical analysis

The method of 2^-ΔΔCt^ was used to analyze the real-time PCR data expressed as the fold change relative to the control group [37]. All data were presented as means ± SEM. Statistical analyses were carried out with SPSS11.0 for windows (SPSS Inc., Chicago, IL, USA). The differences were tested with *t*-test for Independent-Samples. A *P* value of less than 0.05 was considered significant.

## Results

### Body weight and liver weight

As shown in Table 3, chicks that hatched from leptin-treated eggs exhibited significantly lower hatch weight (*P* = 0.000). No alterations were observed in the absolute liver weights, but the liver weight relative to the body weight, or the liver index, was significantly higher in the leptin-treated group at hatching (*P* = 0.018).

**Table 3:** **Effects of*in ovo*leptin administration on body weight, liver weight and liver index (liver weight relative to body weight) in newly hatched chicks**

Parameters	Control	Leptin
Body weight (g)	36.34 ± 0.32	33.30 ± 0.65**
Liver weight (mg)	801.0 ± 22.0	801.7 ± 16.1
Liver index (%)	2.21 ± 0.06	2.42 ± 0.07*

Values are presented as means ± SEM. *P < 0.05, **P < 0.01 compared with control, n = 12.

### Serum and hepatic contents of leptin and hepatic LEPR expression

As shown in Table 4, *in ovo* administration of leptin significantly increased the serum concentration (*P* = 0.009) and liver content (*P* = 0.041) of leptin in newly hatched chicks.

**Table 4:** **Effects of*in ovo*leptin administration on leptin concentration and lipid metabolic parameters in liver and serum of newly hatched chicks**

Parameter		Control	Leptin
Liver	leptin (ng/g T-protein)	42.71 ± 4.06	58.06 ± 5.42*
	TG (μmol/g liver)	23.15 ± 1.38	20.12 ± 0.54
	Tch (μmol/g liver)	16.88 ± 0.62	13.80 ± 0.13**
Serum	leptin (ng/mL)	1.10 ± 0.11	1.53 ± 0.10**
	TG (mmol/L)	0.67 ± 0.06	0.90 ± 0.07*
	Tch (mmol/L)	8.44 ± 0.22	9.93 ± 0.43*
	ApoB (g/L)	0.096 ± 0.009	0.138 ± 0.017*

Values are represented as means ± SEM, *P < 0.05, **P < 0.01 compared with control, n = 12.

**Figure 1:**
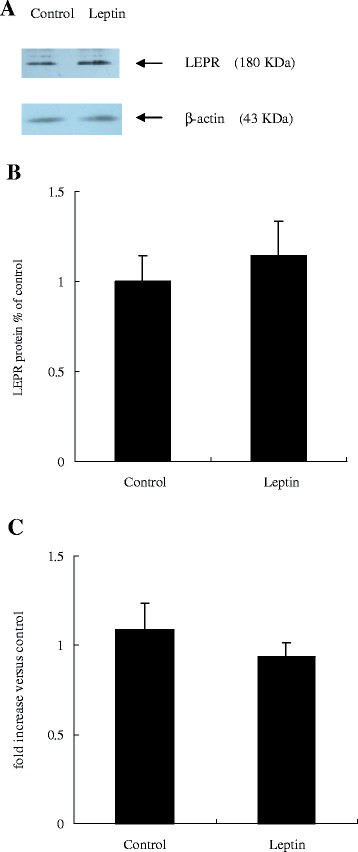
**Effect of*in ovo*leptin administration on hepatic mRNA and protein expression of LEPR in newly hatched chicks. A**: Immunoreactive bands for LEPR and β-actin protein; **B**: LEPR protein content; **C**: *LEPR* mRNA. Values are presented as fold change relative to the control, expressed as means ± SEM, n = 12.

Western blot analysis with a specific antibody against chicken LEPR detected a band of approximately 180 kDa in liver whole cell lysates (Figure 1A and B). *In ovo* leptin injection did not affect either *LEPR* mRNA abundance or LEPR protein content in the liver of newly hatched chicks (Figure 1).

### Lipid contents in serum and Liver

As shown in Table 4, the TG content in the liver was greatly decreased (*P* = 0.053), whereas the TG concentration in the serum significantly increased (*P* = 0.037) in leptin-treated chicks. The hepatic and serum contents of Tch showed the same pattern as TG, being greatly decreased (*P* = 0.001) in liver but increased in serum (*P* = 0.018) of leptin-treated chicks. Moreover, the serum content of ApoB significantly increased (*P* = 0.022) in newly hatched chicks from the leptin-treated group.

### Hepatic expression of genes involved in the regulation of lipid metabolism

**Figure 2:**
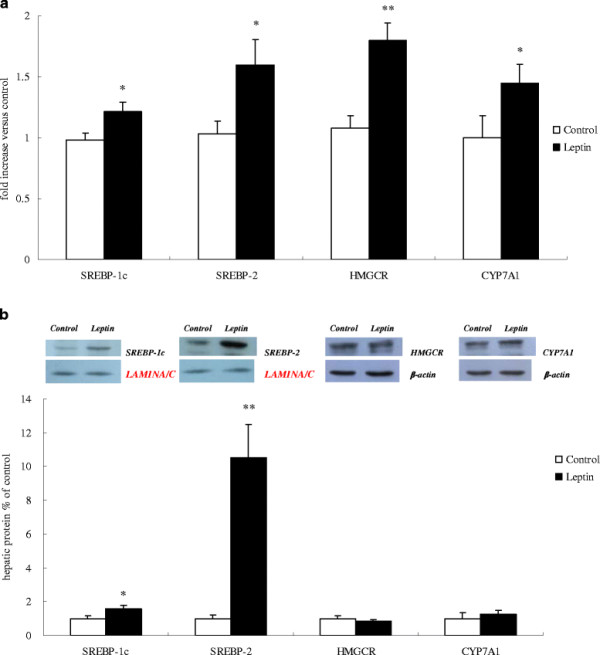
**Effects of*in ovo*leptin administration on hepatic expression of genes involved in lipid metabolism in newly hatched chicks. A:** mRNA; **B:** protein. Values are presented as fold change relative to the control, expressed as means ± SEM. *P < 0.05, **P < 0.01, n = 12.

The hepatic expression of *SREBP-1c* (*P* = 0.027), *SREBP-2* (*P* = 0.030), *HMGCR* (*P* =0.001) and *CYP7A1* (*P* = 0.041) mRNA was significantly up-regulated in leptin-treated chicks at hatching (Figure 2A). In agreement with the mRNA abundances, the protein levels of both SREBP-1c (*P* = 0.032) and SREBP-2 (*P* = 0.001) in liver were significantly increased in leptin-treated chicks. However, the hepatic content of HMGCR and CYP7A1 protein was not affected by *in ovo* leptin treatment (Figure 2B).

### miRNA Quantification

Among the five miRNAs predicted to target *SREBP-1c*, *gga-miR-99a*, *gga-miR-100*, *gga-miR-200b* and *gga-miR-429* were found to be significantly (*P* < 0.05) up-regulated in the liver of leptin-treated chickens. Among the nine miRNAs predicted to target *HMGCR*, the expression of *gga-miR-200a*, *gga-miR-200b* and *gga-miR-429* was significantly increased (*P* < 0.05). It is worth noting that *gga-miR-200b* and *gga-miR-429* were predicted to target both *SREBP-1c* and *HMGCR* (Table 5).

**Table 5:** **Effects of*in ovo*leptin administration on hepatic expression of miRNAs predicted to target*SREBP-1c*and*HMGCR*in newly hatched chicks**

Target Genes	miRNA	Control	Leptin	*P* value
*SREBP-1c*	gga-miR-99a	0.98 ± 0.11	2.11 ± 0.42	0.024
	gga-miR-100	1.00 ± 0.11	2.03 ± 0.38	0.026
	gga-miR-183	1.05 ± 0.23	1.70 ± 0.23	0.070
	gga-miR-200b	0.97 ± 0.11	1.99 ± 0.40	0.033
	gga-miR-429	1.05 ± 0.08	2.04 ± 0.40	0.038
*HMGCR*	gga-miR-15a	1.02 ± 0.12	1.84 ± 0.41	0.086
	gga-miR-15b	1.00 ± 0.10	1.24 ± 0.27	0.427
	gga-miR-16	1.03 ± 0.11	1.62 ± 0.36	0.147
	gga-miR-27b	1.02 ± 0.08	1.22 ± 0.23	0.416
	gga-miR-181a	0.96 ± 0.08	1.00 ± 0.18	0.663
	gga-miR-200a	1.02 ± 0.11	2.52 ± 0.64	0.045
	gga-miR-200b	0.97 ± 0.11	1.99 ± 0.40	0.033
	gga-miR-223	1.04 ± 0.07	1.39 ± 0.28	0.249
	gga-miR-429	1.05 ± 0.08	2.04 ± 0.40	0.038

Values are presented as fold change relative to the control, expressed as means ± SEM (n = 12).

## Discussion

Increasing evidence suggests that maternal leptin programs fetal growth and development in mammalian species. In rats, dams receiving three injections of human recombinant leptin (3.5 mg/kg, Intraperitoneal Injection, i.p.) on days 8, 10 and 12 of gestation produced pups with reduced birth weight [13]. Accordingly, in the present study *in ovo* leptin administration reduced the hatch weight of broiler chickens. This observation, however, contradicts a previous publication in which *in ovo* injection of recombinant mouse leptin on day 5 of incubation advanced hatching by 5 to 24 h and improved the hatch weight of Japanese quails [15]. Perhaps the effect of leptin on Japanese quails is different than its effect on chickens. Furthermore, different doses of a hormone, like leptin, usually have diverse physical effects. These factors may contribute to the discrepancy in the results.

*In ovo* administration of leptin increased the proportional liver weight in the leptin-treated chickens at hatching. The increased liver index could be the consequence of decreased body weight, yet it has been suggested that alterations in proportional liver weight are usually associated with the change in hepatic lipid metabolism in the chicken [38-40]. Our results support this notion as the increased liver index correlated with alterations in hepatic and serum TG and Tch concentrations in newly hatched broiler chickens. Moreover, we observed, for the first time, a positive correlation between liver weight and hepatic leptin production and secretion in prenatally leptin-exposed chickens, which is in agreement with a previous report showing that the liver weight positively correlates with the plasma leptin concentrations in broiler breeder hens [41].

To date, the mechanism by which maternal leptin influences offspring leptin secretion has not been clarified. A transplacental transfer of maternal leptin to the fetus, which increases during late pregnancy in parallel with an up-regulation of expression of the shorter isoforms of the leptin receptor in the placenta, has been reported in rodents [42,43]. It is unknown how exogenous leptin injected into the albumen of the eggs is metabolized and/or transported to the developing embryos. We detected a significant down-regulation of *LEPR* mRNA expression in the yolk sac of leptin-treated embryos at 12 days of incubation (data not shown). However, the physiological significance of this down-regulation remains elusive. It is unlikely that the injected murine leptin remains active over 21 days of incubation, thus the increased hepatic and serum leptin contents in newly hatched chickens are most likely the consequences of an altered developmental program that may occur primarily at an early embryonic stage.

Leptin has been shown to exert direct effects on lipolysis and lipogenesis of adipose tissue in mammalian species [2]. Instead of the mammalian adipose tissue, liver is the primary organ for lipid metabolism in the chicken [17]. In the present study, the TG and Tch contents were greatly decreased in the liver, but were significantly increased in the serum of leptin-treated chickens, which was accompanied by a trend of increase in serum ApoB levels. ApoB is a major protein component of the plasma very low-density and low-density lipoproteins (VLDL and LDL, respectively) and plays an important role in transporting cholesterol and triglycerides from the liver [44]. The associated changes in serum ApoB and TG/Tch may indicate enhanced TG and Tch transport from the liver of leptin-treated chickens at hatching.

SREBPs directly activate the expression of more than 30 genes dedicated to cholesterol and fatty acid synthesis in the liver [18]. In the present study, the mRNA expression and protein contents of SREBP-1c and SREBP-2 were both enhanced in the liver of leptin-treated chicks at hatching. HMGCR and CYP7A1, which are dedicated respectively to the synthesis and uptake of cholesterol, were both significantly up-regulated in the liver of leptin-treated chickens, indicating a positive transcriptional regulation of SREBP-2 on *HMGCR* and *CYP7A1* genes. SREBP-1c and SREBP-2 share a mechanism of feedback regulation, mediated by sterol response elements (SREs) present in the enhancer/promoters of each gene. Accumulation of hepatic TG and Tch has been reported to reduce SREBPs processing [18]. Therefore, the up-regulation of hepatic SREBP-1c and SREBP-2 expression may be the result of the reduced hepatic contents of TG and Tch in leptin-treated chickens.

Leptin could interact with leptin receptor to implement its biological functions [45,46]. Although the serum leptin concentration in the leptin-treated neonatal chicks was significantly increased, no significant alterations were detected in LEPR protein and mRNA expression in liver. Chicken leptin has been reported to desensitize its own response by decreasing the expression of its receptor mRNA [46]. The lack of change in hepatic LEPR expression in leptin-treated chickens implies a possible desensitizing effect of the elevated serum leptin on its receptor. In general, leptin plays an important role in the negative regulation of lipogenesis in mammals [2]. In contrast to its inhibitory effects on the expression of SREBPs [2], HMGCR and CYP7A1 [19] in mammals, *in ovo* leptin administration enhanced mRNA expression of *SREBP-1c/2HMGCR* and *CYP7A1* in the present study, in parallel with elevated serum leptin concentration. It is possible that the effect of leptin on the hepatic expression of lipogenesis genes is species specific, being stimulatory in poultry yet inhibitory in mammals. Taking into consideration the irresponsiveness of hepatic LEPR, another possibility is that the enhanced hepatic expression of SREBPs, HMGCR and CYP7A1 may not be the direct effect of leptin. Other hormones or metabolites, altered by *in ovo* leptin treatment, may participate in the regulation of hepatic lipid metabolism and lipogenic gene expression, as well as hepatic leptin production and secretion.

Recently, considerable interest has been shown towards the role of miRNA in the regulation of lipid metabolism [23,24]. We detected concurrent changes in the expression of miRNAs and their target genes, which seems to contradict the common notion that the expression of miRNAs and their target genes are normally negatively correlated. However, the functions of miRNAs are much more complex and new mechanisms are emerging. For instance, although miRNAs are known to predominantly act in translational repression, *miR-10* has been recently found to bind a group of transcripts containing a terminal oligo-pyrimidine (TOP) motif and to induce their translation [47]. Moreover, a time lag may exist between the expression of miRNAs and the endpoint of their actions in destruction of mRNAs or translational repression of target genes. We observed the uncoupling of *HMGCR* mRNA (up-regulated) and protein (unchanged) in the liver of leptin-treated chicks, which coincided with significantly increased expression of *gga-miR-200agga-miR-200b* and *gga-miR-429* that were predicted to target the chicken *HMGCR* gene. However, further studies are required to clarify whether these miRNAs act as translational repressors to stabilize HMGCR protein content despite increased mRNA abundance.

In the present study, five out of 12 miRNAs predicted to target *SREBP-1c* and/or *HMGCR*, were significantly up-regulated in the liver of leptin-treated chicks. It is noteworthy that *gga-miR-99a* and *gga-miR-100* belong to the miRNA gene family of *miR-99*, while the remaining three miRNAs, *gga-miR-200a*, *gga-miR-200b* and *gga-miR-429*, belong to the miRNA gene family of miR-8, located in the same miRNA cluster. In agreement with our results, it is presumed that miRNAs from the same family share similar biological functions and miRNAs from the same miRNA cluster normally have the same expression pattern. However, further experiments are necessary to validate the function of these five miRNAs on the expression of the target genes and hepatic lipid metabolism in the chicken.

## Conclusions

We have shown for the first time that *in ovo* administration of leptin decreases the hatch weight, and modifies hepatic leptin synthesis and secretion, as well as hepatic lipid metabolism in newly hatched broiler chickens. A miRNA-mediated regulation of cholesterogenic and lipogenic genes, such as *SREBP*s and *HMGCR*, may be involved in these effects.

## Abbreviations

miRNAs = MicroRNAs; LEPR = Leptin receptor; TG = Triglyceride; Tch = Total cholesterol; ApoB = Apolipoprotein B; SREBP-1c = Sterol regulator element binding protein 1c; SREBP-2 = Sterol regulator element binding protein 2; HMGCR = Hydroxy-3-methylglutaryl coenzyme A reductase; CYP7A1 = Cholesterol 7α-hydroxylase 1; SREBPs = Sterol regulatory element binding proteins; ACC = Acetyl CoA carboxylase; CPT-I = Carnitine palmitoyltransferase I; FAS = Fatty acid synthase; PBS = Phosphate buffered saline; SREs = Sterol response elements.

## Competing interests

The authors declare that they have no competing interests.

## Authors’ contributions

YH carried out the experiments, participated in the data collection, data analysis and interpretation, and drafted the manuscript. RZ performed the analysis of the hepatic microRNAs’ expression, helped in data interpretation and paper drafting. YZ and JL performed the protein quantification by western blot analysis. RG provided valuable advice for this study and helped in editing the manuscript. RZ contributed in conception, experimental design, data interpretation and finalized the manuscript. All authors approved the final version of the manuscript for publication.
